# Play face in Japanese macaques reflects the sender’s play motivation

**DOI:** 10.1007/s10071-022-01730-5

**Published:** 2022-12-08

**Authors:** Sakumi Iki, Nobuyuki Kutsukake

**Affiliations:** 1 Center for the Evolutionary Origins of Human Behavior, Kyoto University, Aichi, Japan; 2 Research Center for Integrative Evolutionary Science, Department of Evolutionary Studies of Biosystems, The Graduate University for Advanced Studies, Sokendai, Kanagawa, Japan; 3 Japan Society for the Promotion of Science, Tokyo, Japan

**Keywords:** Japanese macaque, Peer play, Play signal, Play face, Social cognition

## Abstract

Animals often initiate social interactions by exchanging signals. Especially when initiating amicable interactions, signaling one’s friendly stance toward others in advance may be important to avoid being misunderstood as having hostile intentions. We used data on dyadic play fighting in a group of Japanese macaques, *Macaca fuscata*, to examine the function of “play face” at the opening of a play session. We found no support for the previously proposed hypothesis that play face expression is likelier before entering risky situations (e.g., before gaining an undue advantage over the partner) to avoid being misunderstood. The results showed that play face expression was likelier in male juveniles before initiating play with other males than in females before initiating play with males or other females and that juveniles were likelier to express play face before initiating play with others closer in age. As male Japanese macaques play more frequently than females, and juveniles prefer to play with individuals closer in age, play face expression before play initiation may reflect the individual’s motivation for subsequent play interactions. This interpretation is supported by our observation that play bouts lasted longer when initiated with bidirectional play face by both participants than when initiated without play face. We also argued that since there was no tendency that play face was likelier to be expressed toward individuals with low play propensity (e.g., females) or infrequent partners to play with (e.g., individuals more distant in age), Japanese macaques may not tactically deploy this signal to recruit reluctant partners.

**Supplementary Information:**

The online version contains supplementary material available at 10.1007/s10071-022-01730-5.

## Introduction

To initiate a shared activity, animals, including humans, often establish mutual engagement with one another (Gómez 1994; Susswein and Racine 2008). Physical proximity is not sufficient to establish mutual engagement; therefore, animals exchange communicative signals to initiate a shared activity (Genty et al. 2020; Heesen et al. 2021). The opening of an interaction is often accompanied by social signals that signify the characteristics of the emerging interaction. Especially when initiating an amicable interaction, indicating one’s friendly stance toward others in advance may be crucial to avoid being misunderstood as having hostile intentions. Studying how animals initiate social interactions by exchanging signals is important to elucidate how they manage complex social interactions. This study focused on the use and function of facial signals expressed by Japanese macaques, *Macaca fuscata*, when they initiate playful interactions.

Play fighting is a form of social interaction commonly observed in immature mammals (Burghardt 2005), and it involves motor patterns, such as “biting” and “grabbing,” which are seemingly similar to the motor patterns used in serious aggression (Palagi et al. 2016; Špinka et al. 2016). Although these motor patterns are used gently enough to ensure that playmates are not harmed, play fighting can sometimes escalate into overt conflict (Palagi 2018; Iki and Kutsukake 2022). As amicable play interactions can foster social bonds between players (Shimada and Sueur 2018), it is vital for them to avoid unnecessary conflicts. To convey a friendly stance toward their partner and prevent escalation to aggression, animals use a variety of play signals, including facial expressions specific to play contexts (van Hooff 1972; Pellis and Pellis 1996; Palagi et al. 2016).

Studies on play faces and other types of play signals have mainly focused on signals expressed during play sessions rather than those expressed at the start of play (for a few exceptions focused on mutual gaze and gestures at the opening of the play, see Fröhlich et al. 2016; Genty et al. 2020; Heesen et al. 2021). For example, ultrasonic calls by rats (*Rattus norvegicus*) during play decrease the likelihood of play escalation (Kisko et al. 2015; Burke et al. 2017). In dogs (*Canis lupus familiaris*), reciprocal bowing during play serves to sustain play (Palagi et al. 2015). Regarding facial signals, although a study of sun bears (*Helarctos malayanus*) suggested that the frequency and mimicry of play face are not correlated with the duration of the play session (Taylor et al. 2019), other studies have reported that (mimicry of) play face during play prolongs play sessions (chimpanzees: Davila-Ross et al. 2011; western gorillas, *Gorilla gorilla gorilla*: Waller and Cherry 2012; geladas, *Theropithecus gelada*: Mancini et al. 2013; Tonkean macaques, *Macaca tonkeana*: Scopa and Palagi 2016; meerkats, *Suricata suricata*: Palagi et al. 2019).

Although some studies have shown that the opening of a play session is not necessarily accompanied by facial signals (chimpanzees: Spijkerman et al. 1996; Tibetan macaques, *Macaca thibetana*: Wright et al. 2018) and that facial signals may serve to maintain an ongoing play session rather than initiate a new one (geladas: Palagi and Mancini 2011), other studies have suggested that animals initiate play fighting with various kinds of play signals (play bows in dogs: Byosiere et al. 2016; play faces in chimpanzees: Hayaki 1985; gestures in chimpanzees: Fröhlich et al. 2016; also see Heesen et al. 2021). Any investigation of the use and function of facial signals expressed before the first playful physical contact between individuals requires comparing play sessions both preceded and not preceded by facial signals. However, no previous studies have conducted this type of comparison. The current study addressed this issue by focusing on dyadic play fighting in juvenile Japanese macaques.

Japanese macaques display facial expressions (i.e., play face, also called relaxed open-mouth display) that are specific to the context of play by opening the mouth in a relaxed way and drawing the corners of the mouth slightly backward (Preuschoft and van Hooff 1995; Petit et al. 2008; Scopa and Palagi 2016). Owing to their morphological and functional similarities, this expression is considered homologous to human laughter (van Hooff 1967, 1972; de Waal 2003; Davila-Ross et al. 2015). This study compared dyadic play fighting sessions preceded by play face with ones not preceded by play face. Our key goal was to establish the situations in which play face is displayed and how play face at the opening of play fighting affects subsequent interactions. Play faces are sometimes expressed bidirectionally by both individuals before the first playful physical contact (see Supplementary Information 1.1 and 1.2). Whether both or only one individual expresses play face in the opening of a play bout might affect how subsequent interactions proceed. Hence, we also addressed whether a functional difference exists between bidirectional and unidirectional play face before the opening of a play fighting bout. Specifically, we tested the following two hypotheses. Note that these hypotheses are not necessarily mutually exclusive while some predictions derived from them are incompatible (i.e., *Prediction 1b* vs. *Prediction 2b*).

### Hypothesis 1

The expression of play face reflects an individual’s motivation to play.

It has been suggested that play face represents the spontaneous expression of an individual’s internal state, such as a playful propensity and pleasurable emotion (van Hooff 1972; Demuru et al. 2015; Scopa and Palagi 2016). Hence, we hypothesized that play face expression before play fighting initiation is likelier in individuals with higher play frequency and with preferable partners. In Japanese macaques, males play more frequently than females (Eaton et al. 1986; Nakamichi 1989), and they prefer to play with other males (Glick et al. 1986). Therefore, we predicted that play face expression before the start of play fighting would be likelier in a male initiating play with another male than in an individual comprising a pair of other sex combinations (*Prediction 1a*). Several primate studies have also suggested that individuals prefer to play with partners of a similar age and body size (long-tailed macaques, *Macaca fascicularis*: Fady 1976; Japanese macaques: Mori 1974; chimpanzees: Mendoza-Granados and Sommer 1995; western gorillas: Maestripieri and Ross 2004; Palagi et al. 2007; rhesus macaques, *Macaca mulatta*: Kulik et al. 2015). Hence, we predicted that individuals would be likelier to express play face before the first playful contact with playmates of a similar age (*Prediction 1b*). In addition, if play face indexes the expresser’s willingness to play, then the play bouts preceded by play face will last longer than those not preceded by play face. Therefore, we predicted that a play fighting bout would last longer when initiated with a bidirectional play face by both players than with a unidirectional play face by one of the players and that a play bout would last longer when initiated with a unidirectional play face than without (*Prediction 1c*).

### Hypothesis 2

Play face is expressed before engaging in a risky situation.

Play signals have been suggested to be used in risky situations with the potential for overt conflict, because individuals can express their playful and non-harmful stance toward playmates through play signals (Bekoff 1972; Matsusaka 2004; Palagi 2009). Since play fighting involves aggressive actions, it is expected that communicating one’s benign intention to the playmate is particularly important in this type of play. In Japanese macaques, the inter-player difference in dominance rank and age may affect the escalation of play fighting into serious conflict, as playful attacks from individuals of higher rank and greater age than their partners are likelier to trigger a negative response (e.g., screaming) by relatively lower-ranked or younger playmates during a play bout (Iki and Kutsukake 2022). Hence, we predicted that an individual of a higher rank (*Prediction 2a*) or older age (*Prediction 2b*) than their partner would be likelier to express play face before the beginning of play fighting to avoid provoking a negative response in the partner. Moreover, play fighting is not only amicable but also has a competitive aspect, as participants in play fighting compete to gain an advantage over their partners by attacking them unilaterally (e.g., Aldis 1975; Biben 1986; Bauer and Smuts 2007). A player may be likelier to express play face before gaining an undue advantage over the partner to avoid provoking a negative response. Therefore, we predicted that the proportion of time during which a player maintains an advantage would be greater in a bout preceded by the player’s play face than in a bout not preceded by play face (*Prediction 2c*).

## Materials and methods

### Study sites and subjects

This research was conducted at Jigokudani Monkey Park in Shiga-Heights, Nagano Prefecture, Japan. One of us (S.I.) conducted behavioral observations from July to October 2018 and from July to September 2019. The total observation time was approximately 1008 h. The study group was a provisioned group of free-ranging Japanese macaques. The demographic records of the group, including information on maternal kinship, have been kept by the Park’s staff since 1962. Since mature male Japanese macaques move between groups, and since some individuals are on the group’s periphery (Sprague et al. 1998), we could not determine a precise number of individuals in the group; however, the group comprised approximately 240 individuals. In September 2019, the group comprised 82 adult females (> 4 years of age), approximately 20 adult males (> 4 years of age), 110 juveniles (1–4 years of age), and 32 infants (< 1 year of age). The group was fed barley, soybeans, and apples four times daily (0900, 1200, 1500, and 1630) by the staff.

### Data collection

We estimated the dominance ranks by observing unidirectional agonistic interactions between adult females using ad lib sampling methods. We considered a unidirectional agonistic interaction to have occurred when individual A approached individual B and B expressed submissive behavior (e.g., a bared-teeth display) or fled or when A unilaterally attacked B. Since the Japanese macaque is a highly despotic species, most agonistic interactions are completely unidirectional (Thierry 2000). Japanese macaques form linear dominance hierarchies (Chaffin et al. 1995), so we indexed dominance using an ordinal rank based on the outcome of these interactions: we assigned an ordinal rank value of 1 to the highest-ranked adult female, a value of 2 to the next-highest-ranked female, and so on. Since the maternal dominance rank is socially transmitted from mother to offspring (Chapais 1988; Kutsukake 2000) in Japanese macaques, we considered immature individuals to have the same rank as their mothers. Overall, we recorded 1112 unidirectional agonistic interactions.

We collected data on play fighting by having the observer stand in specific positions in the park so that almost all individuals of the group could be observed. The observer recorded all visible play fighting sessions between juveniles using a digital video camera (HDR-TD10 211; Sony, Tokyo, Japan). The observer regularly changed the observation location to avoid observation bias. We did not adopt focal sampling because it is not efficient for infrequent behaviors, such as play fighting (Martin and Bateson 2007). Moreover, we avoided observing the animals for 30 min before and 30 min after the provisioning time. Several studies have suggested that play frequency increases before the feeding period (Palagi 2007; Norscia and Palagi 2011). Hence, avoiding observations around the feeding time might have decreased the sample sizes. However, during the feeding period, individuals may interrupt a play bout to acquire the food in front of them. Since we focused on the play bout durations, we decided not to collect data around or during the feeding time.

Following the procedures used in previous studies on play fighting in Japanese macaques (Reinhart et al. 2010; Iki and Hasegawa 2021), play bouts were required to meet the following requirements: only two individuals were involved; the entire bout took place on relatively flat ground and not in a three-dimensional environment, such as trees; the individual did not use objects (e.g., stones and branches); and at least one play bite occurred without any negative expressions (e.g., screaming and bared-teeth displays). We excluded cases in which individuals continuously transitioned from grooming, contact-sitting, mounting, or chasing to play fighting.

A total of 578 play bouts met these requirements. We investigated the function of the play face before a play bout began by focusing only on cases in which both players faced each other when they initiated play and in which the faces of both players could be seen clearly in the video data. Although only a limited camera angle provided a clear view of the faces of both individuals, 118 bouts met the video requirements. As several studies have shown that Japanese macaques begin to show signs of sexual maturity as early as 4 years of age (e.g., Hamada and Yamamoto 2010; Pflüger et al. 2019), we excluded bouts involving 4-year-old individuals from the analysis. The remaining 113 bouts involved 62 individuals (see Table 1 for detailed information). Each individual was involved in a mean of 3.65 bouts (SD: 2.85; range 1–11). Of the 113 bouts, 86, 21, and 6 involved male–male, male–female, and female–female dyads, respectively. Regarding age differences, 72, 30, and 11 bouts were between dyads with age differences of 0, 1, and 2 years, respectively.

**Table 1:** Overview of the individuals in the dataset

Observation year	Age (years)	No. of males	No. of females
2018	1	7	3
	2	14	6
	3	4	2
2019	1	17	5
	2	5	2
	3	8	3

### Video coding

We used the ELAN software (Lausberg and Sloetjes 2009) to conduct frame-by-frame video analyses (30.3 FPS). We defined the beginning of each bout as the moment at which an individual directed any playful attack (i.e., biting, grabbing, or wrestling) toward their partner and the end as the time when the players stopped playing for at least 10 s. We defined a play bout as initiating with a play face if one or both individuals expressed play faces immediately before the first playful physical contact (i.e., within 5 s). No physical contact or any other type of facial expression was made between the play face expression and the first playful contact. Since only a limited camera angle provided a clear view of the facial expressions of both individuals, determining which of the two first opened its mouth was often not possible when both individuals expressed play faces. Hence, we did not focus on the question of which individual was the first emitter. Following previous studies (Biben 1986; Bauer and Smuts 2007; Iki and Hasegawa 2020), we considered a player to have the advantage when he/she pinned down or attacked the partner unidirectionally. A player was considered to have pinned down the partner if the player stood or sat with their weight on the partner, causing the partner to lie down in a lateral, supine, or prone position. A player was considered to have attacked the partner unidirectionally when they bit or grabbed the partner without being bitten or grabbed by the partner. Overall, the mean duration of 113 play bouts in our dataset was 24.76 s (SD = 29.57). In terms of play face expression before play initiation, 56, 28, and 29 bouts were preceded by bidirectional, unidirectional, and no play face, respectively. Twelve randomly chosen play bouts (i.e., 10.6% of all play bouts) were coded by a separate rater. We calculated Cohen’s kappa and observer accuracy to assess inter-rater reliability using the KappaAcc software (Bakeman 2022). The resulting values of Cohen’s kappa and observer accuracy were 0.72% and 90% for the direction of play face expression (i.e., bidirectional, unidirectional, or none), respectively, and 0.69 and 87% for the state of play every 0.1 s (i.e., whether one of the players held an advantage, the players attacked each other, or did not attack each other), respectively. The values of Cohen’s kappa indicated that inter-rater reliability showed substantial agreement (Landis and Koch 1977), and the values of observer accuracy were above the threshold of 85%, as proposed by Bakeman (2022).

### Statistical analyses

We analyzed the data using generalized linear mixed models (GLMMs; the “glmer” function in the lme4 package) in R ver. 4.1.2 (R Core Team 2021). We set our alpha level to 0.05.

We analyzed play face expression by running a GLMM with a binomial error structure and a logit link function. We conducted this analysis by labeling each player in the dyad as a “subject player” and its playmate as a “partner.” The response variable was whether the play face was expressed by the subject player before the first physical contact. We included the following key predictors as fixed effects: the sex combination between the subject player and their partner (categorical: male–male, male–female, female–male, female–female; relevant to *Prediction 1a*); the rank difference between players (continuous; relevant to *Prediction 2a*); the age difference between players (continuous; relevant to *Prediction 2b*); and the absolute value of the age difference (continuous: relevant to *Prediction 1b*). We controlled for possible confounding effects by including the following factors as control variables: subject player’s age (continuous); subject player’s absolute dominance rank (continuous); and kinship between players (categorical: kin or non-kin). Individuals were considered kin if they were maternal siblings. This kinship criterion was set with reference to a study by Chapais et al. (1997), who showed a threshold for nepotism in Japanese macaques. We dealt with pseudoreplication by including the subject player identity, play partner identity, dyad, and play bout as random effects.

We analyzed the duration of the play bout by running a GLMM with a gamma error structure and a log link function. This analysis was conducted at the dyadic level. We included the direction of the play face before a bout began (categorical: bidirectional, unidirectional, or none) as a key predictor (relevant to *Prediction 1c*). We controlled for possible confounding effects by including the following factors as control variables: the sex combination of the dyads (categorical: male–male, male–female, female–female); the absolute value of the rank difference between players (continuous); the absolute value of the age difference between players (continuous); and kinship between players (categorical: kin or non-kin). We included the identity of the dyad as a random effect.

We analyzed the proportion of time during which a player maintained an advantage using a GLMM with a gamma error structure and a log link function. We conducted this analysis by labeling each player in the dyad as a “subject player” and its playmate as a “partner.” The response variable was the total duration during which a subject player had an advantage in a bout. We used the play bout duration (log-transformed) as an offset variable. We included the direction of the play face before a bout began (categorical: bidirectional play face, unidirectional play face only by a subject player, unidirectional play face only by a partner, or no play face) as a key predictor (relevant to *Prediction 2c*). As control variables, we included the following factors: the rank difference between players (continuous); the age difference between players (continuous); the absolute value of the age difference (continuous); kinship between players (categorical: kin or non-kin); and the sex combination between a subject player and their partner (categorical: male–male, male–female, female–male, female–female). We included the identity of the subject player, play partner, dyad, and play bout as random effects.

We fitted all possible combinations of fixed effects and compared the Akaike information criterion with a correction for small sample size (AICc) scores using the “dredge” function in the MuMIn package in R. The model with the lowest AICc score was considered the best model (i.e., the model that provided a satisfactory explanation of the variation in the data), and models with a difference of < 2 between the model’s AICc score and that of the best model (ΔAICc) were typically considered to have levels of statistical support similar to the best model. We dealt with this uncertainty in model selection by employing a multi-model inference method (Burnham and Anderson 2002). Using the set of models with ΔAICc < 2, we calculated the model-averaged coefficients and 95% confidence intervals (CIs) with the “model.avg” function in the MuMIn package. The model-averaged coefficients were standardized by setting the “beta” argument in the “model.avg” function to “partial.sd.” We also evaluated the importance of variables by calculating the sum of the Akaike weights over models with ΔAICc < 2 containing each variable (Burnham and Anderson 2002). These procedures enabled us to estimate the strength of the relationship between each explanatory variable and the response variables while simultaneously considering the relative likelihood of each model. We analyzed the play bout duration by conducting Tukey’s post hoc multiple comparison with the model with the lowest AICc to examine the differences among the three categories of the direction of play face (i.e., bidirectional, unidirectional, or none). To check the proportion of total variance explained by the best models, we calculated the conditional *R*^2^ for the models with the lowest AICc values using the “r.squaredGLMM” function in the MuMIn package.

## Results

**Fig. 1:**
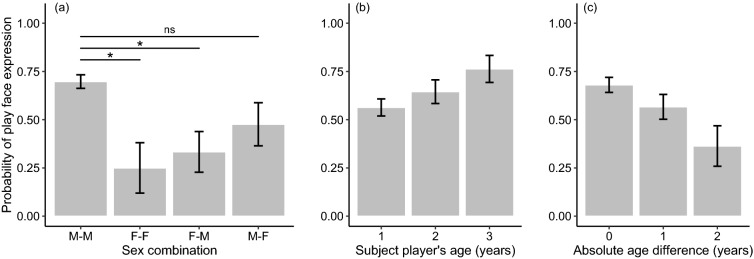
The mean probability of the expression of play face according to the **a** sex combination between a subject player and a partner, **b** a subject player’s age, and **c** absolute age difference between players. Error bars represent standard errors. Sample size: *N* = 226. *p < 0.05; *ns* non-significant

We found four models with ΔAICc < 2 for the probability of play face expression (see Supplementary Information 2). The ΔAICc of the null model was 16.77. The conditional *R*^2^ for the model with the lowest AICc was 0.57. The multi-model inference analysis identified the sex combination between players as one of the variables with the strongest effect. Its variable importance reached the maximum value (1.0), and the 95% CI of its standardized coefficient did not overlap with zero (Table 2), indicating that the expression of play face was significantly likelier in a male before initiating play with another male than in a female before initiating play with a male or another female (Fig. 1a). No significant difference was detected in the probability of play face expression between dyads combining male subject players and female partners and male–male dyads (Fig. 1a). These results partially support *Prediction 1a*. The variable importance of the subject player’s age reached the maximum value, and the 95% CI of its standard coefficient did not overlap with zero (Table 2), indicating that older players would be likelier to express play face (Fig. 1b). Also, the variable importance of the absolute value of the age difference between players reached the maximum value, and the 95% CI of its standardized coefficient did not overlap with zero (Table 2). This result indicated that the probability of play face expression increased as the absolute value of the age difference decreased, reaching its maximum value when the age difference was zero (*Prediction 1b* supported; Fig. 1c). The results also showed that the rank and age differences between players had no significant effects on the probability of play face expression (*Predictions 2a* and *2b* not supported; Table 2).

**Table 2:** Details of the model-averaged coefficients

	Sum of weights	Standardized coefficient	Lower 95% CI	Upper 95% CI	*p*-value
Probability of play face expression					
Sex combination (ref: male–male)	1.00				
Female–female		− 0.719	− 1.298	− 0.141	0.015
Female–male		− 0.496	− 0.930	− 0.062	0.025
Male–female		− 0.200	− 0.607	0.206	0.334
Absolute age difference	1.00	− 0.584	− 1.137	− 0.030	0.039
Subject player’s age	1.00	0.656	0.171	1.142	0.008
Age difference	0.22	− 0.169	− 0.530	0.192	0.359
Rank difference	0.18	− 0.127	− 0.495	0.240	0.498
Subject player’s rank	0.17	0.139	− 0.303	0.581	0.539
Play bout duration					
Direction of play face (ref: none)	1.00				
Bidirectional		0.345	0.155	0.536	< 0.001
Unidirectional		0.121	− 0.068	0.310	0.211
Absolute age difference	1.00	− 0.269	− 0.480	− 0.058	0.013
Sex combination (ref: male–male)	0.71				
Female–female		0.205	0.018	0.392	0.032
Female–male		− 0.036	− 0.237	0.165	0.727
Kinship (ref: kin)	0.68				
Non-kin		− 0.198	− 0.418	0.022	0.078
Proportion of the duration of advantage					
Sex combination (ref: male–male)	1.00				
Female–female		0.291	− 0.097	0.679	0.141
Female–male		0.002	− 0.383	0.388	0.990
Male–female		0.532	0.150	0.915	0.006
Rank difference	0.38	− 0.261	− 0.631	0.109	0.167
Kinship (ref: kin)	0.36				
Non-kin		− 0.288	− 0.791	0.215	0.262
Absolute age difference	0.31	− 0.274	− 0.717	0.170	0.226
Direction of play face (ref: none)	0.21				
Bidirectional		0.272	− 0.068	0.612	0.117
Unidirectional (partner)		− 0.077	− 0.339	0.184	0.563
Unidirectional (subject player)		− 0.053	− 0.306	0.199	0.678
Age difference	0.08	0.033	− 0.323	0.389	0.262

Sample size: *N* = 226, 113, and 226 for the probability of play face expression, play bout duration, and proportion of the duration of advantage, respectively

**Fig. 2:**
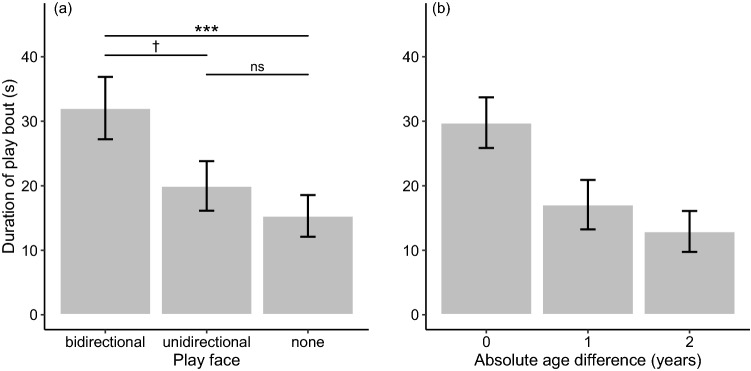
The mean duration of a play bout according to the **a** direction of play face and **b** absolute age difference between players. Error bars represent standard errors. Sample size: *N* = 113. ***p < 0.01; †*p* < 0.1; *ns* non-significant (Tukey’s post hoc test)

We found three models with ΔAICc < 2 for the duration of play bouts (see Supplementary Information 2). The ΔAICc of the null model was 15.88. The conditional *R*^2^ for the model with the lowest AICc was 0.46. The multi-model inference analysis showed that the direction of play face had the strongest effect, as the 95% CI of its standardized coefficient did not overlap with zero, and its variable importance reached the maximum value (Table 2). Post hoc tests showed that play bouts initiated with a bidirectional play face lasted significantly longer than ones not initiated with a play face (Tukey’s HSD: *p* < 0.001) and tended to last longer than ones initiated with a unidirectional play face (Tukey’s HSD: *p* = 0.068), whereas the duration of play bouts initiated with a unidirectional play face was not significantly different from the duration of play bouts initiated with no play face (Table 3; Fig. 2a; *Prediction 1c* partially supported). The analysis also showed that the play bout duration was significantly affected by the absolute value of the age difference between players (Table 2), indicating a shorter duration of a play bout between pairs with a larger age difference (Fig. 2b).

**Table 3:** Results of Tukey’s post hoc multiple comparison tests of the effect of direction of play face on play bout duration

	Estimates	SE	*Z*	*p*-value
None < bidirectional	− 0.876	0.235	− 3.720	< 0.001
None < unidirectional	− 0.347	0.259	− 1.339	0.373
Bidirectional > unidirectional	0.529	0.238	2.222	0.068

For the Tukey tests, we used the function “emmeans” in the R package emmeans

**Fig. 3:**
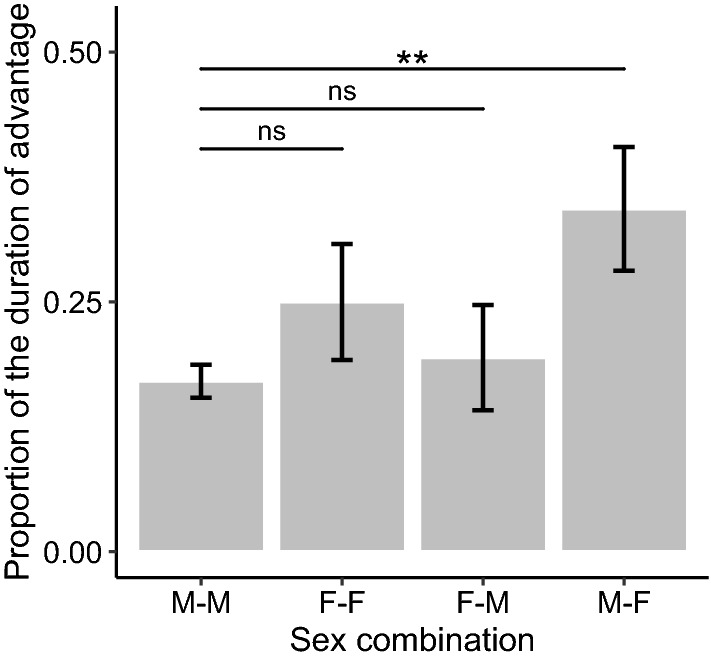
The mean proportion of time during which a subject player maintained an advantage according to the sex combination between a subject player and a partner. Error bars represent standard errors. Sample size: *N* = 226. **p < 0.01; *ns* non-significant

We found 12 models with ΔAICc < 2 for the proportion of time during which a subject player maintained an advantage (see Supplementary Information 2). The ΔAICc of the null model was 2.62. The conditional *R*^2^ for the model with the lowest AICc was 0.88. The multi-model inference analysis showed that the sex combination between players was the variable with the strongest effect. Its variable importance reached the maximum value, and the 95% CI of its standardized coefficient did not overlap with zero (Table 2). This result indicates that a male player gained a greater proportion of the advantage over a female partner than over a male partner (Fig. 3). The results also showed that the play face direction had no significant effects (Table 2; *Prediction 2c* not supported).

## Discussion

Overall, our results provided substantial support for *Hypothesis 1* (i.e., that play face expression reflects an individual’s motivation for the subsequent interaction), but not for *Hypothesis 2* (i.e., that play face is expressed before engaging in a risky situation). We showed that play face expression was likelier in a male juvenile before initiating a bout with another male than in a female juvenile before initiating a bout with another female or a male (Fig. 1a). Although no significant difference was detected in the likelihood of play face expression between a male initiating play with another male and a male initiating play with a female, these results imply that play face expression was likelier in males than in females (*Prediction 1a* partially supported). The analysis also indicated that Japanese macaque juveniles would be likelier to express play face before initiating play with others closer in age than with others more distant in age (*Prediction 1b* supported; Fig. 1c). Considering that play frequency is higher in males than in females and that juvenile Japanese macaques prefer to play with individuals of the same age (Mori 1974; Eaton et al. 1986; Glick et al. 1986; Nakamichi 1989), our results suggest that play face before the first playful physical contact is likelier to be expressed by an individual with a higher play frequency (i.e., a male) who is initiating play with a preferable partner (i.e., individuals closer in age). The finding that a play bout initiated with a bidirectional play face lasted longer than a bout not initiated with play face and tended to last longer than a bout initiated with a unidirectional play face (Fig. 2a; *Prediction 1c* partially supported) also supports this interpretation.

Relevant to *Hypothesis 1*, it is interesting whether play face functions to invite and/or reengage reluctant partners to play. Our finding that play face expression is likelier in individuals with higher play frequency (i.e., males) and with frequent partners (i.e., same-age peers) suggests that play face is not tactically deployed to recruit reluctant and/or infrequent partners (e.g., females and individuals distant in age) but is automatically expressed by an individual who is engaged in a bout that is about to take place. Also, if play face motivates a reluctant partner to play, it would be expected that bouts initiated with unidirectional play face would last longer than bouts initiated without play face, but we found no such result. To elucidate whether play face in Japanese macaques is an unintentional emotional expression or is deployed tactically, future studies need to examine, for example, whether individuals can adjust their play face expressions in response to the attentional state of the partner or whether individuals tend to use facial expressions repeatedly when they do not get the desired response from the partner (Demuru et al. 2015).

Researchers have hypothesized that play face expression is likely before entering a risky situation to avoid escalation into overt conflict (e.g., Bekoff 1972), but our results did not support this hypothesis (i.e., *Hypothesis 2*). We predicted that play face expression before the first physical playful contact would be likelier in relatively higher-ranked or older players whose playful attacks can sometimes trigger a negative response (e.g., screaming) in the partner (Iki and Kutsukake 2022) than in lower-ranked or younger partners. However, we found no effect of rank or age difference on the probability of play face expression (Table 2; *Predictions 2a* and *2b* not supported). Also, we found no evidence that the presence of play face before the start of a play bout is related to the proportion of the duration of an advantage during the play bout (Table 2; *Prediction 2c* not supported). Several studies suggest that play signals have the function of communicating that the aggressive action the sender of the play signal is about to deliver has a benign, not hostile, intent (play face in Hanuman langurs, *Semnopithecus entellus*: Špinka et al. 2016; play bows in dogs: Bekoff 1995) and function to prevent play escalation (ultrasonic calls in rats: Burke et al. 2017). Although our results are seemingly inconsistent with these previous studies, more detailed research is needed to determine whether play faces in Japanese macaques have these previously proposed functions. One example of how to examine this is to compare the use of play face between play fighting and less competitive social play not involving biting (Demuru et al. 2015), although we focused only on play fighting involving at least one biting. Such a comparison would allow us to test whether play face expression is likelier before starting play fighting, which incurs a higher risk of escalation, than before other kinds of less competitive social play. Furthermore, although we analyzed only play bouts that did not escalate into serious conflict, it is also crucial to directly test whether play face in Japanese macaques functions to prevent play escalation.

Although we did not make any specific prediction relevant to this result, we found that males held a greater proportion of an advantage over female partners in a bout than over male partners (Fig. 3). Some researchers have proposed that juvenile play fighting develops physical strength and skills for real fighting and contributes to dominance acquisition in later life stages (e.g., Hass and Jenni 2010; Pellegrini and Smith 1998; Briffa and Lane 2017; but see Sharpe 2005). Since mature male Japanese macaques leave the natal group and move to other groups (Sprague et al. 1998), their ranking in the natal group as juveniles is inconsistent with their ranking in their new group as adults (Suzuki et al. 1998). Nevertheless, juvenile play in Japanese macaques may be related to their species-specific dominance structure. From this perspective, our finding that juvenile males held a greater advantage over female partners than over male partners may relate to the fact that adult male Japanese macaques typically rank higher than females (Johnson et al. 1982). Also, although Japanese macaque males do not necessarily obtain a higher ranking through aggressive interactions with other males (Suzuki et al. 1998; Takahashi 2002), male–male play fighting may tend to be competitive and, therefore, a male player may have difficulty holding an advantage for long periods of time when playing with another male.

Note that caution must be taken in generalizing the results of this study to other species/groups or other types of play for several reasons. First, the use and function of play signals may differ in species with an egalitarian/tolerant social style that contrasts with the extremely despotic social style of Japanese macaques. Some studies suggest that more egalitarian/tolerant species have more complex communication systems due to the need to manage complicated social relationships (Freeberg et al. 2012; see also Kavanagh et al. 2021). Second, it is also worth noting that our study focused only on dyadic play fighting. Previous studies have suggested that polyadic play fighting sessions are characterized by higher unpredictability (Cordoni et al. 2018), and the probability of play face expression is higher in polyadic sessions than in dyadic sessions (Palagi 2008). While this study did not provide substantial support for the hypothesis that play face expression is likelier before engaging in risky situations, we cannot exclude the possibility that individuals did not necessarily have to use play face because dyadic sessions were sufficiently predictable. Japanese macaque juveniles may need to express play faces when initiating polyadic play fighting characterized by high unpredictability. To examine this possibility, future studies must compare play face expression between dyadic and polyadic sessions. Third, Japanese macaques are known to have intraspecific variations in the level of tolerance (Nakagawa 2010; Kaigaishi et al. 2019). Since our study relies on a limited sample size (*N* = 113 bouts) from a single group, caution should be taken in generalizing our results to other groups of Japanese macaques. Nonetheless, we hope this study will provide a foundation for comparison with other more tolerant groups of Japanese macaques.

In conclusion, whereas previous studies have mainly focused on the play signals used during ongoing play sessions, we have focused on the use and function of facial signals expressed before the start of play fighting. Our results that play face expression at the opening of play in Japanese macaques is likelier in individuals with higher play frequency (i.e., males) and with frequent partners (i.e., same-age peers) imply that this type of facial signal indexes the sender’s motivation for subsequent interactions, but may not function to invite reluctant or infrequent partners.

## Supplementary Information

Below is the link to the electronic supplementary material.

Videos of play bouts in which play faces are expressed bidirectionally by both individuals before the first playful physical contact. Supplementary file1 (MOV 23731 KB)

Videos of play bouts in which play faces are expressed bidirectionally by both individuals before the first playful physical contact. Supplementary file2 (MOV 18830 KB)

Details of the models with ΔAICc < 2. Supplementary file3 (XLSX 13 KB)

## Data Availability

The data sets analyzed in this study can be accessed at https://bit.ly/3SWklWG
